# Influence of Cognitive Neural Mechanism on Music Appreciation and Learning

**DOI:** 10.1515/tnsci-2019-0010

**Published:** 2019-04-23

**Authors:** Yang Lv

**Affiliations:** 1 School of Humanities, Xidian University, Xi'an 710126, China

**Keywords:** Cognitive Neural Mechanism, Influence of cognitive neural system, Music appreciation and learning

## Abstract

Based on the related research results of the relationship between cognitive neural mechanism and music in recent years. In this paper, we study the relationship between the cognitive neurons and music from the overlapping and separation of brain neuro-mechanism and the significance of functional relationships between the two. Through analysis, it can be seen that the cognitive neural mechanism has a certain influence on music appreciation and learning and the studies on brain-damaged patients show that the two may have separate and independent neural bases. Finally, we find the influence of sub-consciousness on decision making through the measurement of SCRs (skin conductance responses), and thus propose a decision model modified by subconscious and make an outlook for future research trends.

## 1 Introduction

In today’s Chinese society, with the development of the economy, more and more children join the amateur learning music team, for example, participate in choir, piano training classes, violin training classes. Many parents believe that music learning can improve children’s cognitive ability and make them smarter. Music training has thus become one of the effective means of improving cognitive ability.

The question is, is music training relevant to cognitive ability and may improve people’s cognitive ability? Or, to what extent does music training improve cognitive ability? As early as 1993, Nature magazine reported on the relationship between music listening and spatial cognition. The study found that after listening to Mozart’s first movement in the D major double piano sonata (K448) in 10 minutes, the spatial reasoning ability of college students was short-lived improvement. This phenomenon is known as the “Mozart effect.” Although some subsequent studies have validated the existence of the Mozart effect, some studies have found that there is no correlation between listening to music and spatial cognition. Recently, Voracek and Formann used meta-analytic methods to analyse data from existing studies. The results show that the “Mozart effect” does not exist. Perhaps the time spent listening to music is too short, and the relationship between music listening and spatial ability is not stable enough. So, can long-term music training affect cognitive ability? Throughout the research of the past ten years, many scholars have done a lot of work in this field.

**Figure 1:**
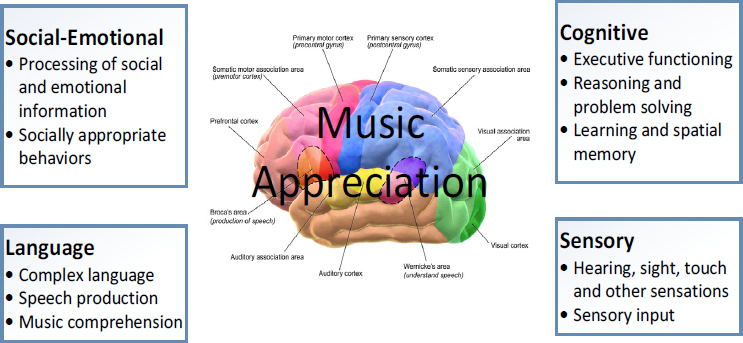
Cognitive nervous system related to music appreciation and learning

With the advancement of cognitive neuroscience and the further improvement of the level of brain activity measurement instruments, the application of cognitive neuroscience has also rapidly developed in social, psychological, economic, cultural, commercial and other fields, drawing attention from relevant disciplines ^[1, 2, 3]^. At present, there are interdisciplinary areas where with clearly defined academic terms, such as neurosociology, neuro-economics and neuromarketing. The cognitive neural mechanism related to music appreciation and learning is shown in Figure 1.

Among the cognitive abilities of human beings, language ability is the most important one that has been studied together with music and has been used to compare with music. Compared with other human cognitive abilities, language and music are improvised. Their related structures are developed in time. They are structurally similar and are organized by certain elements according to certain rules ^[4, 5, 6]^ and spoken language has characteristics similar to the pitch in music, which reaches the human perception system in the form of frequency spectrum. Many similarities between language and music make the two objects of comparison for researchers. We can also see many connections between the two in terms of research results of cognitive neuroscience. In this paper, the authors mainly compare the cognitive neural mechanisms of the two from findings of cognitive neurological researches and discuss the functional relationship between the two from cultural significances.

## 2 Cognitive nervous system

### 2.1 The overlap of cognitive neural mechanisms

Cognitive neuroscience research aims to clarify the brain mechanism of cognitive activity, which is, how the human brain invokes its various levels of components, including molecules, cells, brain tissue regions, and the whole brain to achieve various cognitive activities. Some branches of traditional neuroscience, such as neuropsychology, psychophysiology, physiological psychology, neurobiology, and behavioural pharmacology, have absorbed the theory of cognitive science and new techniques of neuroscience, gradually forming cognitive neuropsychology. Various branches of cognitive neuroscience have exist, such as learning, cognitive psychophysiology, cognitive physiology psychology, cognitive neurobiology, and computational neuroscience. Since the beginning of the late 1980s, cognitive neuroscience research has made remarkable progress in a short period of time, and has a tremendous impact on the theoretical construction of traditional cognitive psychology and developmental psychology and the research in various content areas. Cognitive development research is no exception. Because cognitive developmental psychology and the development of neuroscience science are interested in many common problems, the development of cognitive neuroscience is gaining more and more attention and becoming the most One of the popular cross-research areas.

Studies in China and abroad show that the primary auditory cortex is activated when it responds to language and music in the same way; The secondary auditory cortex is activated when listening to and understanding words and listening to musical scales, sound images and melody; In understanding language representations and reading scores, the supramarginal gyrus involves in processing; A f-MRI comparative study of melody processing and word processing shows that both stimuli activate the gyri temporales transversi, anterior temporal lobe and planum temporale ^[7]^; The results of the direct comparative study by Chinese scholars Li Enzhong, et al. using news and music clips as stimuli show that both stimuli have activated superior frontal gyrus and superior temporal gyrus (not Wernicke’s area) at both sides, etc. The overlapping of the neural mechanism of music and language, on the one hand, provides the physiological and neurological evidences for understanding the relationship between music and language, and on the other hand, expands the previous research on the functional areas of the brain and provides us with a more comprehensive and detailed understanding of functional areas of the brain ^[8]^.

### 2.2 The separability of cognitive neural mechanism

In cognitive neuroscience, the dual separation method is mainly used for the localization of brain function and the study of complex cognitive function systems. The experimental logic is based on the double dissociation, but has been modified to accommodate cognitive neurological experiments. The experimental logic is as follows:

In the brain function localization, there are patients A and B, and the damaged brain parts are different, respectively X and Y, so that both patients can perform two cognitive tasks *a* and *b*. If A completes a, it cannot be completed. b, B completes task b and cannot complete task a, then X brain is related to a cognitive function, and Y brain is related to b cognitive function.

The example of the brain mechanism separation experiment on the first floor is also correct, but it does not mean that both patients have to complete the tasks *a* and *b*.

The experimental tasks are not necessarily only *a* and *b*, and can perform multiple tasks; there are not only two subjects, but also multiple subjects with different brain regions can participate in the experiment.

**Figure 2:**
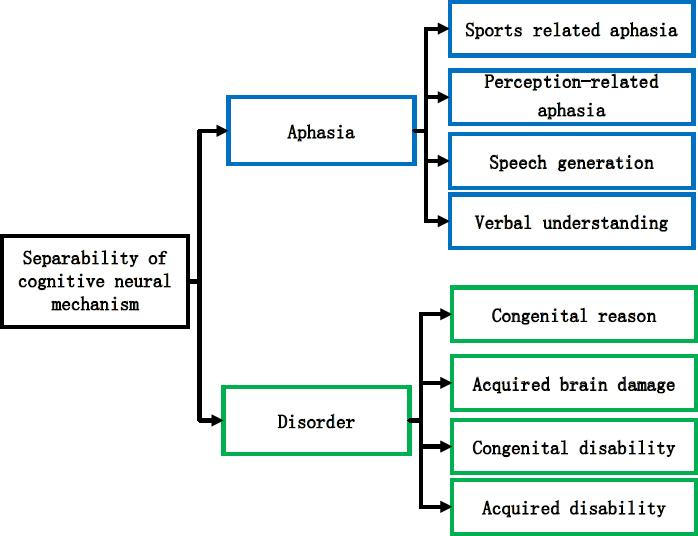
Reparability of cognitive neural mechanism

The research evidences on the overlap of the neural mechanisms of music and language continue to increase, but this connection does not enable us to conclude that music and language share a common neural mechanism. The studies on the separation of the neural mechanisms of music and language cognition originate from two aspects, on the one hand, studies on the music and language processing of normal people and on the other hand, studies on brain-damaged patients ^[9]^. The specific contents of the separation process of cognitive neural mechanism are shown in Figure 2.

The study on brain-damaged patients mainly includes two special groups: aphasia and amusia sufferers. Aphasia refers to a clinical syndrome in which a patient suffers from an abstract signal thinking disorder due to a lesion in the central nervous system and loses the ability to express in spoken language, words and comprehension. It is generally divided into two categories: movement and perception, which involve speech production and speech comprehension. Amusia is the loss of the ability to understand music and perceive a certain component of music due to congenital causes or acquired brain damage. An amusia sufferer is not impaired in auditory system and can perceive everyday sounds, but cannot perceive melody and cannot perceive the wrong tone in familiar melody. Amusia is generally divided into congenital and acquired ones ^[10]^. Amusia sufferers may not suffer from aphasia and vice versa, which indicates that music cognition and language cognition systems do not overlap completely and there may be independent encephalic regions.

## 3 Music appreciation and learning

### 3.1 The structural rules of music

**Figure 3:**
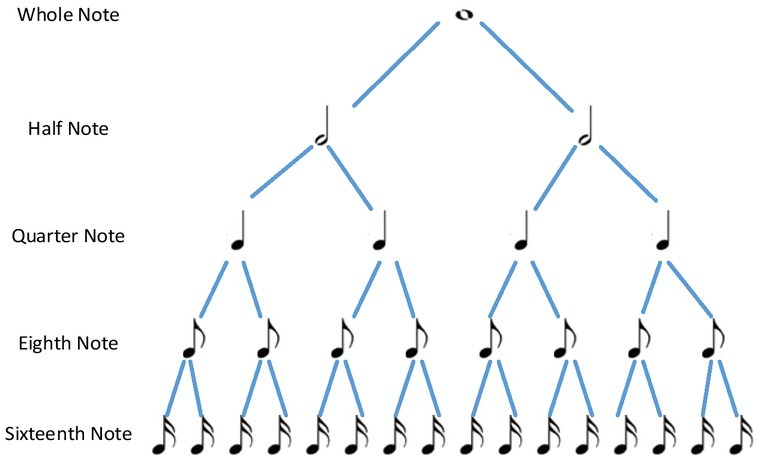
The hierarchy in music

Syntax refers to the principle followed by the separation of elements to form a sequence, including multiple levels, such as word formation, rules of phrase formation and rules of sentence building in the language and rules of chords, chord-sequence and musical scales in music. See Figure 3.

Syntax enables the brain to transform the input information so that these separate elements grouped together in hierarchical relationships can convey specific information ^[11]^. In language, the general form of expression of information through syntax is “who did what to whom”; while in music, the information conveyed through syntax is reflected by rhythm variation between tension and relaxation. These diverse rules are stored in the human mind in an implicit form, allowing people to perceive incongruous situations, such as subject-verb disagreement in a sentence or the out-of-tune chords in music. According to music theory, harmony has a structural function that limits the form of music: it organizes the vertical integration of pitch, establishes or modulates mode and tonality, develops or terminates a structure; and listeners can anticipate the subsequent harmonic characteristics according to the previous harmony.

### 3.2 The meaning of music appreciation and learning

The ability of language perception involves not only the perception of speech, but also the individual’s understanding of words and passages. Many studies have examined the relationship between music training and speech perception. Related studies have shown that not only children’s musical pitch ability is positively correlated with phoneme awareness, but also rhythm ability is related to phoneme awareness and phonological awareness. Escalda, Lemos and França found that music-trained children performed better in phonological awareness tasks than children without music training. The speech perception advantage formed by music training is also supported by cognitive neuroscience research. For example, children trained in music have a greater MMN amplitude for the processing of vowel/a/time and start time variability compared to children without music training; time and brainstorm speech coding for preschool children’s music training There is a significant positive correlation; White-Schwoch et al. found that in terms of syllable/da/induced brainstem neural activity latency, subjects with longer musical training duration were shorter than those without music training and music training, and then The difference between the two is not significant. Researchers believe that early and long-term music training will gradually change the subcortical auditory function, and this change may last a lifetime. Even in the context of noise, the contribution of music training to brainstalk speech coding can be verified in preschool children, school-age children, young people, and middle-aged and older people. Tracking studies further illustrate that music training contributes to language perception.

Overy found that through 15-week music learning, the phonological awareness of children with dyslexia was significantly improved. Similarly, Degé and Schwarzer randomly assigned 41 pre-school children to three groups of music, speech or sports training. After 20 weeks of training, children who received music and voice training scored significantly improved on the phonological awareness test, while children who received exercise training did not improve their scores. At the same time, there was no difference between the scores of the music training group and the voice training group. The results of this study show that, like speech training, music training can promote speech perception.

The promotion of music training to language perception has also been verified by other studies. On the other hand, research shows that there is also a link between music training and language understanding. In terms of vocabulary understanding, existing studies have mainly used the similarity and vocabulary test in the Wexler Intelligence Scale to explore. Schellenberg found that through 36 weeks of music training, 144 children scored significantly higher on the class-like test than those who did not. This result was verified by subsequent studies. In the vocabulary test, relevant research shows that the length of music training can predict the scores of the vocabulary test. Indeed, children who have been trained in music have better vocabulary comprehension than children who have not received music training. Tracking studies validated the impact of music training on children’s vocabulary understanding. Whether it is 36-week music performance (keyboard or vocal) training or a 4-week music listening training, children who have been trained in music have a higher vocabulary comprehension than non-music training children. The above studies further show that music training can promote vocabulary understanding.

In terms of paragraph comprehension, Corrigall and Trainor examined the relationship between the length of music training for children aged 6-9 and the ability to understand paragraphs. The results of the study show that although the influence of music listening perception score, word decoding score, IQ and weekly reading time is still significantly positively correlated with reading comprehension ability, it controls age and parental education. After the level and the age at which the child begins to learn music, the correlation between the two is close to a significant level. Indeed, Moritz et al. also found that preschool children’s musical rhythm ability was not able to predict their paragraph comprehension in their second year. Tracking studies on children with language impairments showed that through 6 weeks of music rhythm training, the reading comprehension of children with dyslexia was improved. Through 4 weeks of music training, the subjects with poor verbal ability were also significantly better in paragraph comprehension improved. However, there are differences in the findings of normal human paragraph understanding: although 8-week or 4-week music training does not improve children’s paragraph comprehension, Schellenberg found that one-year music training can promote children’s paragraph comprehension effect. The inconsistency in the above findings may be due to differences in music training time.

From these studies, it can be seen that for normal children, short-term music training may not improve their paragraph comprehension, and at least one year of music training may promote paragraph comprehension. In summary, studies have shown that music training can promote individual understanding of speech, vocabulary and paragraphs to a certain extent. This stimulating effect of music training may be due to the sharing of cognitive resources or neural mechanisms by music and language processing, making music skills likely to migrate to the language domain.

Expression of meaning and exchange of information is the most basic function of a language, but it is still not clear whether music can convey meaning. A lot of linguists believe that music does not have the function of expressing specific meanings ^[12]^, but music theorists believe that composers express their thoughts through music and the meaning conveyed by music is an integral part of music.

The meaning of music can be defined from at least four aspects: imitating an object in the form of a specific sound (the sound of a certain tone is close to the sound of a certain object); a particular mood caused by the music; associations beyond the music aroused; and the emotional induction evoked by different rhythmic changes in the music. In the research results, especially in the processing of syntax, music processing either activates similar encephalic regions or evokes similar event-related potentials (ERPs), which means that music may have some of the same neural basis.

## 4 Modeling the Influence of Cognitive Neural System on Music Appreciation and Learning

### 4.1 Model analysis

**Figure 4:**
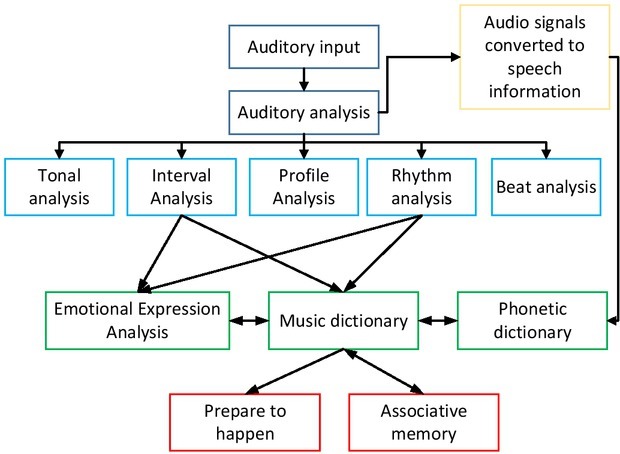
Music processing model

Peretz, et al. proposed a model of brain music processing through neuropsychological investigations of various types of amusia sufferers. Music processing includes the processing of basic modules and the processing of connection between modules. Some modules, such as encoding of tunes and analysis of melodic contours, are considered to be exclusively for music processing and some modules are shared by music and language. However, the model still needs support from more well-defined amusia cases and fMRI data. The process of music processing is shown in Figure 4.

Each box in the figure represents a processing module and the arrows represent the association among these processing modules and the flow of information. Brain damage can damage the function of the processing modules themselves (boxes) and may also damage the information connection

among the processing units (arrows). The solid black box indicates that the processing unit is exclusive to music processing. It is unclear whether the 3 processing units of “emotional expression analysis”, “rhythm analysis” and “beat analysis” in italics are exclusive to music processing.

### 4.2 Data analysis

The two major methods of cognitive neuroscience related experiments are: functional magnetic resonance imaging (fMRI) and event-related potentials (ERP) measured by EEG. The relationship between them is shown in Table 1.

**Table 1:** Comparison of two cognitive neuroscience related experimental methods

	fMRI	ERP
Basic principles	Changes in blood flow in the brain and changes in the magnetic field of oxygenation of haemoglobin with high spatial precision, but the scanning time is in seconds and it is impossible to accurately record brain activity in shorter time	The neurons in the brain area are discharged with high time accuracy. Only the brain surface electrical signals are recorded and it is difficult to trace the source accurately.
Lab environment	The subjects lay flat in a claustrophobic space, with high magnetic fields, high psychological pressure, and few repetitions of experiments.	Ordinary quiet area, relaxed and stress-free, repeated test times, if you only measure EEG, you can measure as needed in any field environment
Popularity	High price, inconvenience, large size, fixed	Cheap, easy to use, small size, portable
Easily accessible	Easy to get image results of active brain areas, analysis is relatively simple	Not easy to get a significant waveform

The introduction of cognitive neuroscience in the field of music is clearly to further uncover the fundamental mysteries of music appreciation and learning, that is, to study the inner mechanisms of the brain’s ability to produce some kind of music appreciation and to reveal the “black box” between the outside stimulus or the introspection and the behaviour. In terms of research methods, the cognitive neural mechanism follows the experimental characteristics of neurobiology and further inherits the central idea of experimental economics. The main content is to observe and measure the basic rules of human brain activity in making music decisions and to discover the neural mechanism of a certain type of economic behaviour. The experimental model of cognitive neural mechanism and music appreciation is shown in Table 2.

**Table 2:** Experimental Model of Cognitive Neural System and Music Appreciation

	Traditional music appreciation	Cognitive nervous system	Music Appreciation and Learning Under the Influence of Cognitive Nervous System
Hypothesis	Complete rationality or bounded rationality under conditional constraints	Limited rationality of behavioural level	Nerve-level bounded rationality
Mode	Individual preference decision behaviour	Mind decides behaviour	Brain mechanism determines musical behaviour
Variable	Deterministic model without sensory variables	Sensory variables, combined with cognitive	Measuring sensation, cognitive quantification
Decision processing	Controllable processing	Controllable processing, emphasizing the role of cognitive systems	Controllable and automatic processing, interactions between cognitive and affective systems

**Figure 5:**
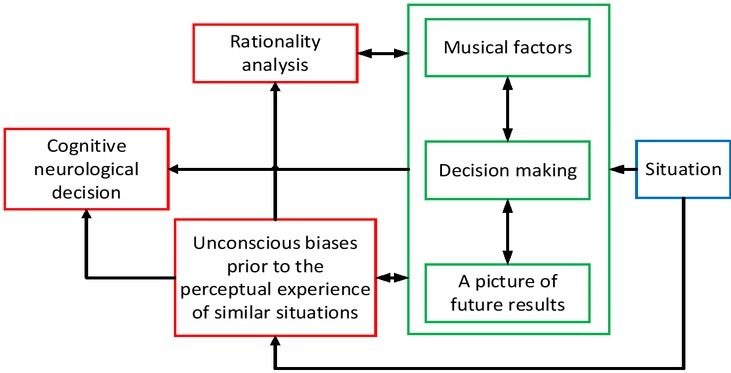
Cognitive nervous system decision process for music appreciation and learning

In this paper, through the measurement of SCRs (skin conductance responses), the authors find the influence of sub-consciousness on decision making and thus propose a decision model modified by subconscious. In addition to “rationality strategies” and “rational choices for future results” and other factors affecting decision making, “unconscious bias (subconscious) before similar situational perceptions” also influences decision making through two types of approaches: the first is to influence decision making indirectly through “rationality strategies” and “rational choices for future results” and the second is to influence decision making directly. And these processes of influence are confirmed by behavioural research combined with measurement of changes in SCRs. The cognitive neural mechanism decision-making process of music appreciation and learning is shown in Figure 5.

**Figure 6:**
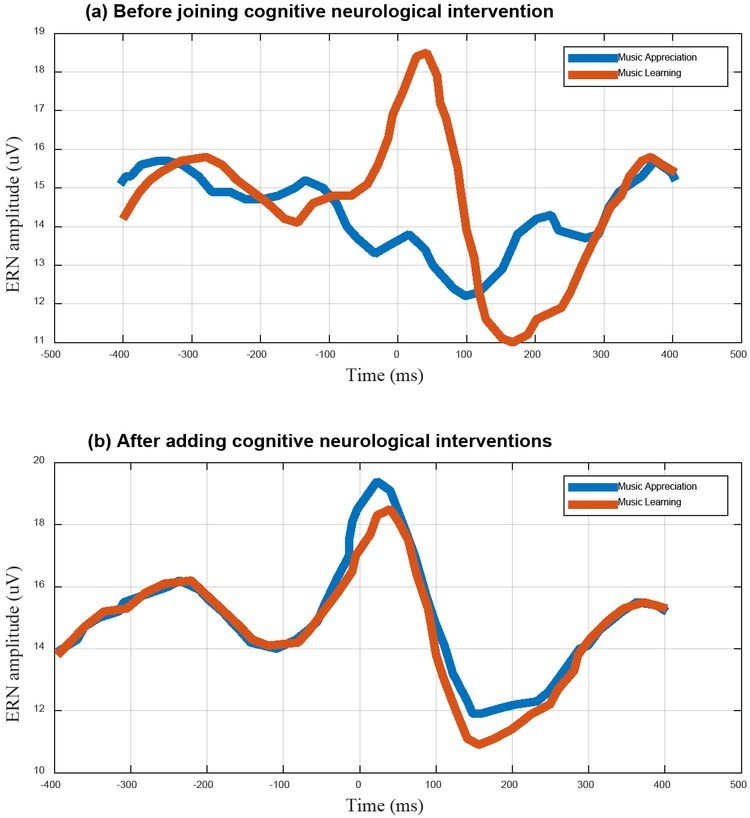
The Influence of Cognitive Nerve System on Music Appreciation and Learning

In order to better study the model of influence of cognitive neural mechanism on music appreciation and learning, we use event-related potentials (ERPs) measured by EEG to analyse the error related negativity (ERN) in the anterior cingulate cortex region of the brain during music appreciation. In this study, we find that the effects of cognitive neural mechanism have a greater effect on music appreciation and learning and the ERN resulted is more intense. The specific results are shown in Figure 6.

## 5 Conclusions

In this paper, we study the relationship between the cognitive neurons and music from the overlapping and separation of brain neuro-mechanism and the significance of functional relationships between the two. Through analysis, it can be seen that the cognitive neural mechanism has a certain influence on music appreciation and learning and the studies on brain-damaged patients show that the two may have separate and independent neural bases. Finally, we find the influence of subconsciousness on decision making through the measurement of SCRs (skin conductance responses), and thus propose a decision model modified by subconscious and make an outlook for future research trends.
